# Micropatterning of Metal Nanoparticle Ink by Laser-Induced Thermocapillary Flow

**DOI:** 10.3390/nano8090645

**Published:** 2018-08-22

**Authors:** Sewoong Park, Jinhyeong Kwon, Jaemook Lim, Wooseop Shin, Younggeun Lee, Habeom Lee, Hyun-Jong Kim, Seungyong Han, Junyeob Yeo, Seung Hwan Ko, Sukjoon Hong

**Affiliations:** 1 Optical Nanoprocessing Lab, Department of Mechanical Engineering, Hanyang University, 55 Hanyangdaehak-ro, Sangnok-gu, Ansan, Gyeonggi-do 15588, Korea; woong93@hanyang.ac.kr (S.P.); limjaemook@hanyang.ac.kr (J.L.); caribou11@hanyang.ac.kr (W.S.); twilit77@hanyang.ac.kr (Y.L.); 2 Applied Nano and Thermal Science Lab, Department of Mechanical Engineering, Seoul National University, 1 Gwanak-ro, Gwanak-gu, Seoul 08826, Korea; jhs0909k@snu.ac.kr (J.K.); habeom.lee@snu.ac.kr (H.L.); 3 Surface Technology Group, Korea Institute of Industrial Technology, 156 Gaetbeol-ro, Yeonsu-gu, Incheon 21999, Korea; hjkim23@kitech.re.kr; 4 Department of Mechanical Engineering, Ajou University, San 5, Woncheon-Dong, Yeongtong-Gu, Suwon 16499, Korea; sy84han@ajou.ac.kr; 5 Novel Applied Nano Optics Lab, Department of Physics, Kyungpook National University, 80 Daehak-ro, Pook-gu, Daegu 41566, Korea; junyeob@knu.ac.kr; 6 Institute of Advanced Machinery and Design (SNU-IAMD), Seoul National University, Gwanak-ro, Gwanak-gu, Seoul 08826, Korea

**Keywords:** selective laser sintering, metal nanoparticle ink, laser-induced thermocapillary flow

## Abstract

Selective laser sintering of metal nanoparticle ink is a low-temperature and non-vacuum technique developed for the fabrication of patterned metal layer on arbitrary substrates, but its application to a metal layer composed of large metal area with small voids is very much limited due to the increase in scanning time proportional to the metal pattern density. For the facile manufacturing of such metal layer, we introduce micropatterning of metal nanoparticle ink based on laser-induced thermocapillary flow as a complementary process to the previous selective laser sintering process for metal nanoparticle ink. By harnessing the shear flow of the solvent at large temperature gradient, the metal nanoparticles are selectively pushed away from the scanning path to create metal nanoparticle free trenches. These trenches are confirmed to be stable even after the complete process owing to the presence of the accompanying ridges as well as the bump created along the scanning path. As a representative example of a metal layer with large metal area and small voids, dark-field photomask with Alphabetic letters are firstly created by the proposed method and it is then demonstrated that the corresponding letters can be successfully reproduced on the screen by an achromatic lens.

## 1. Introduction

The development of novel manufacturing processes for nanomaterial is important to match rapidly increasing number of portable and personalized devices, while these processes can vary in great extent according to the constituent nanomaterials and target applications. These nanomaterials are firstly synthesized and processed through bottom-up approaches [1], but nanoscale structures can be prepared through different methods as well [2,3]. Regarding the processing methods, a wide range of solution processes have been developed to leverage the strengths of nanomaterials [4], while cost-effectiveness and scalability of the manufacturing process are also becoming important [5]. At the same time, various processes are under development to enhance or protect the properties and the functions of the target nanomaterial [6,7].

Among various nanomaterials, metal nanoparticle (NP) has received large amounts of attention as it enables facile preparation of metal layer on versatile substrates through simple low-temperature sintering process [8,9]. The sintering of metal NP ink can be conducted by numerous techniques [10,11,12], yet selective laser sintering (SLS) [13,14,15,16,17] has a number of advantages over other methods. In the previous studies on SLS process for metal NP ink, a focused laser is utilized as a micro heater based on the photothermal reaction which induces melting and sintering of metal NP layer locally. Since the reaction occurs only at the laser focus, patterned metal layer is readily created by scanning the laser beam using motorized stage [14,18] or Galvano mirrors [16,17]. The SLS process is also confirmed to be compatible with heat-sensitive flexible substrate [15,16] and stretchable elastomers [19] given that the laser parameters are carefully controlled. Thanks to these advantages, the SLS of metal NP ink has been investigated extensively to date in order to expand applicable NPs [20,21,22] and potential applications [14,17,18].

While on the other hand, the disadvantage of SLS process is also clear. SLS process is often not suitable for metal patterns with high pattern density since it is a direct writing method in principle, i.e., the focused laser beam has to scan the entire designated metal layer. As a result, the scanning time increases linearly to the density of the metal pattern. In this regard, selective laser ablation (SLA) of metal NP is reported as a complementary process for the metal layer that largely consists of metal area [23,24], yet a pulsed laser at nanosecond or shorter pulse width is required for efficient SLA process. Considering that a continuous-wave (CW) laser is utilized for SLS process in general [15,16,21], an entirely different optical platform is necessary to conduct SLA process, which increases the overall cost as a manufacturing process. As a solution to these limitations, we introduce laser patterning of metal NP ink based on the thermocapillary flow induced by a focused laser. While the previous studies on SLS process for metal NP ink creates the metal pattern along the scanning path, the metal NPs are rather pushed away in the proposed patterning process. The proposed method is different from SLA process since the NPs are not ablated but only rearranged, whereas the entire process can be conducted using the identical platform that performed the previous studies on SLS process for metal NP ink. Its potential as a patterning technique is further validated through the reproduction of Alphabetic letters on the screen using photomasks created by the proposed method.

## 2. Materials and Methods

As a representative metal NP ink, silver (Ag) NP ink was used throughout the study. Ag NP ink was purchased (NPS-J, Harima Chemicals, Inc., Tokyo, Japan) and used without further purification. For a typical experiment, Ag NP ink was firstly deposited on a glass substrate (Microscope slides at 1 mm thickness, Marienfeld) at 500 rpm for 300 s using a spin-coater, (ACE-200, Dong Ah Trade Corp., Seoul, Korea) while the spin-coating duration was varied when investigating the effect of the drying condition. 532 nm continuous-wave (CW) diode-pumped solid-state (DPSS) laser (Sprout-G-5W, Lighthouse Photonics, San Jose, CA, USA) uses Nd:YVO_4_ as its gain medium, while its spatial mode is TEM_00_ at M^2^ = 1.0 − 1.1 with the beam diameter of 2.3 mm ± 10%. The laser was focused with a 5× objective lens (M Plan Apo 5×, Mitutoyo, Kawasaki, Japan) on the Ag NP layer, and the sample was scanned using a motorized 2-axis translational stage (ANT130-060-XY-25DU-XY-CMS-MP-PLUS, Aerotech, Pittsburgh, PA, USA) along the programmed scanning path. For the laser scanning, the laser power and the scanning speed were controlled in the ranges of 0.10 ~ 0.50 W and 0.1 ~ 10.0 mm/s respectively. In order to transform the remaining Ag NP ink into continuous metal layer, the sample underwent bulk heating on a hot plate (MSH-30D, Daihan Scientific, Wonju, Korea) at 200 °C for 2 h. The transmission and reflection images were taken by an optical microscope (BX53M, Olympus, Tokyo, Japan). Atomic Force Microscopy (AFM) and Scanning Electron Microscope (SEM) together with Energy Dispersive X-ray (EDX) analysis were conducted using an NX-10 from Park Systems and a JSM-7600f from JEOL (Tokyo, Japan), respectively.

## 3. Results and Discussion

**Figure 1:**
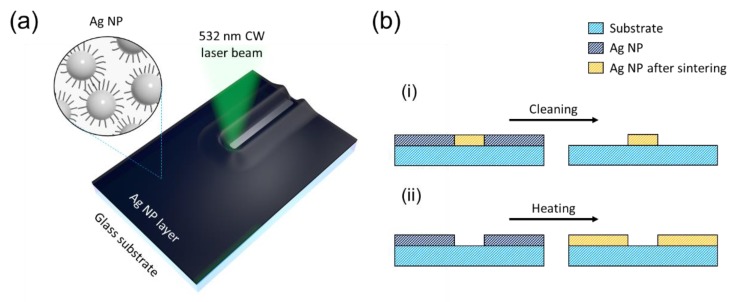
(**a**) Schematics of the proposed laser micropatterning for Ag NP ink; (**b**) The process flow of (i) the previous studies on SLS process for Ag NP ink and (ii) the proposed method.

The previous studies on SLS process for Ag NP ink has been regarded as an additive manufacturing process, seeing that the metal pattern is created along the laser scanning path. On the other hand, the laser-induced thermocapillary effect utilized in the proposed study results in opposite process which removes the Ag NP ink from the laser scanning path as schematically illustrated in Figure 1a. The thermocapillary force can be expressed as the following formula: [25]

(1) $$\tilde{\tau }\cdot \hat{n}=\nabla \sigma =\frac{\partial \sigma }{\partial T}\nabla T,$$

where $\tilde{\tau }$, $\hat{n}$, σ and T are the shear stress, the surface normal, the surface tension and the temperature, respectively. Since the surface tension of a solvent decreases with the temperature in many cases (∂σ/∂T<0), the thermocapillary force acts towards the direction of negative temperature gradient, i.e., away from the center of the focused laser spot [26]. Although the temperature coefficient of surface tension (∂σ/∂T) is small for the current ink (*n*-tetradecane: −0.0869 × 10^−3^ N/m·K) [27], the thermocapillary shear flow can be critical when coupled with the extremely high temperature gradient induced by the focused laser which is estimated to be several tens of K/μm at the current configuration [25,28]. We have confirmed that the resultant laser-induced Marangoni shear flow can create Ag NP free trenches at microscale width when the laser parameters are carefully controlled, analogous to the previous studies on the laser-induced thermocapillary dewetting of polymer thin films [25]. Figure 1b summarizes the process flow diagram for the previous studies on SLS process for metal NP ink and the proposed laser micropatterning method. The procedure of the previous SLS for Ag NP ink, although there may exist some variations, can be summarized as the following steps: (1) Ag NP ink is firstly coated on the target substrate, mostly through wet-processing such as spin-coating or blade coating. (2) Ag NP ink layer coated on the target substrate is exposed to a focused laser beam in order to initiate photothermal reaction for localized sintering of Ag NP ink to create continuous Ag electrode in a selective manner. The exposure is usually incorporated with scanning scheme to create the Ag layer at the designated pattern. (3) The remaining Ag NP ink which is not subject to the sintering should be removed afterwards. The removal of these Ag NPs is usually achieved by simple cleaning of the entire sample with the solvent [15,16]. In the proposed process, additional heating step is conducted instead of cleaning after the laser scanning in order to transform distinct Ag NP ink into the continuous conductive metal layer. These two laser processes very much resemble the characteristics of positive and negative photoresist in the photolithography process. When exposed to light, two photoresists show different results upon applying the developer: the positive photoresist is removed, while the negative photoresist remains. In this regard, the proposed method is similar to the positive photoresist to some extent, seeing that the Ag NP ink is removed by the exposure to a focused laser beam.

It is noticeable from Section 2 that the overall process flow before the heating step is almost identical to the previous studies on SLS process for metal NP ink except for the relevant experimental parameters, and we found that the spin-coating condition is the most critical parameter to initiate the proposed laser patterning process. In the previous studies on SLS process for metal NP ink, the laser-induced thermocapillary effect has been regarded as a byproduct to the laser processing which should be avoided [13]. As a consequence, the spin-coating duration is set to be sufficiently long [16] so that the solvent of the Ag NP ink is completely dried, leaving a fully plasticized film in order to suppress the laser-induced Marangoni flow to a negligible level. In this regard, the amount of laser-induced thermocapillary effect can be rather enhanced by changing the spin-coating conditions.

**Figure 2:**
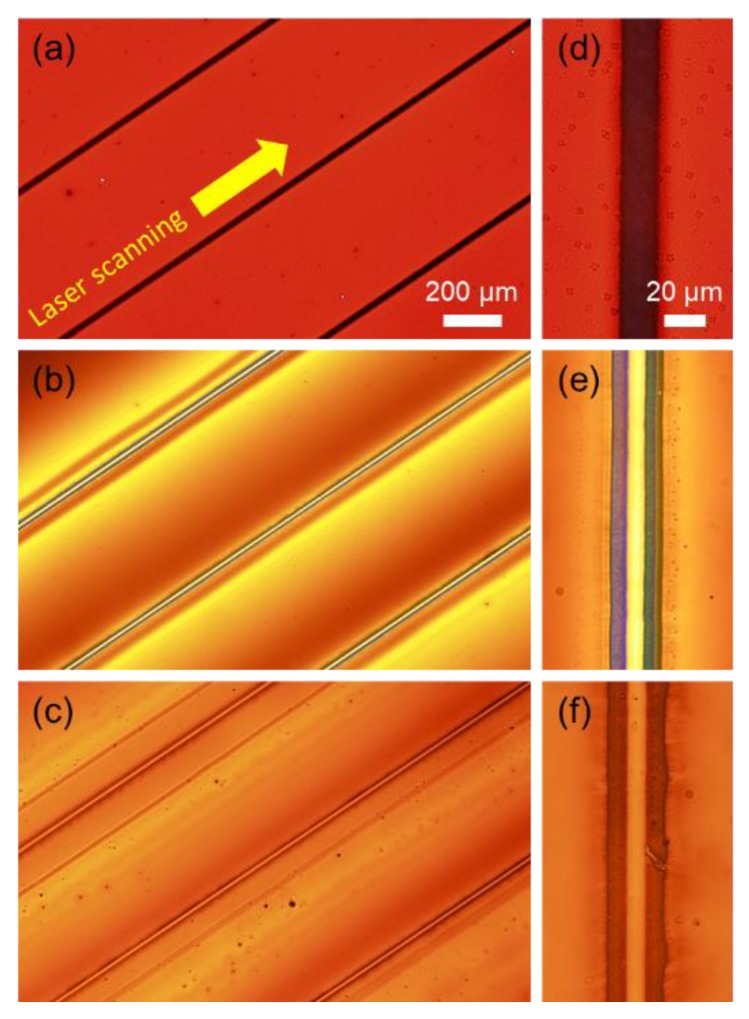
Transmission optical microscope images at (**a**–**c**) low magnification and (**d**–**f**) high magnification immediately after the laser scanning procedure on the Ag NP layer prepared at different spin-coating duration of (**a**,**d**) 1800 s (**b**,**e**) 300 s and (**c**,**f**) 60 s. The rotation speed has been fixed to be 500 rpm in every case.

Figure 2a shows the transmission optical microscope image of the Ag NP ink that corresponds to the fully plasticized case (spin-coating at 500 rpm for 1800 s) immediately after the laser scanning at 400 mW and 1 mm/s along the line patterns at equal spacing. It is observable that the optical transmittance is dropped along the scanning path as the Ag NPs are melted and coalesced by the focused laser to create highly conducting and reflective layer, yet there is no clear indication of thermocapillary flow even in the high magnification image (Figure 2d). The laser-induced thermocapillary effect becomes evident when the spin-coating duration is decreased to 300 s at the same rotation speed as shown in Figure 2b,e, which are the optical images of the Ag NP ink after conducting the identical laser scanning procedure. In this case, the optical transmittance is rather higher at the center along the scanning path due to the removal of Ag NP ink by the thermocapillary shear flow. At the same time, accompanying ridge emerges at the lateral edges of the laser scanning path presumably due to the displacement and subsequent sintering of Ag NPs swept out from the trench [25]. Meanwhile, further decrease in the spin-coating duration may fail the proposed patterning process as shown in Figure 2c,f. Different from Figure 2b,e, the optical transmittances along the laser scanning path and the remaining Ag NP ink are almost equivalent, implicating that the Ag NP ink in the vicinity of the scanning path replenishes the trench created by the laser-induced thermocapillary flow.

Given a fixed spin-coating condition, the resultant morphology of the Ag NP ink layer after the scanning is then highly dependent on the laser scanning parameters. Since the metal content of the Ag NP ink used in the current study (63.7 wt %, data provided by the supplier) is sufficiently high, not only laser-induced thermocapillary shear flow from the solvent, but also substantial amount of sintering between the constituent Ag NPs may occur upon the laser scanning. Since the crucial factors for the sintering and the thermocapillary shear flow are the temperature and its gradient respectively, the balance between these two parameters determines the final morphology of the Ag NP ink layer.

**Figure 3:**
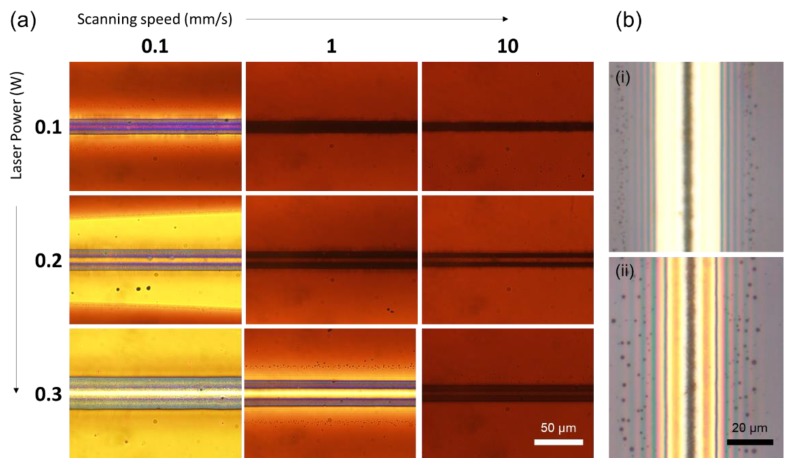
Combinatorial study for optimum laser power vs laser scanning speed. (**a**) Transmission optical microscope images of the Ag NP layer after conducting the laser scanning at 0.1 ~ 0.3 W laser power and 0.1 ~ 10 mm/s scanning speed; (**b**) Reflection optical microscope image of the Ag NP layer (i) immediately after scanned at 0.3 W laser power and 1 mm/s and (ii) after stored in the ambient condition for 10 min.

Figure 3a summarizes the representative outcomes of the Ag NP ink layer immediately after conducting the laser scanning at various scanning speed and laser power, captured by transmission optical microscopy at the same illumination condition. At 0.1 W laser power, the laser scanning fails in separating the Ag NP ink layer to expose the surface of the substrate at any scanning speed. At higher laser powers, the Ag NP ink layer starts to share common characteristics, having two distinct lines created by a single scanning. Upon the scanning, the temperature rise in the lateral direction should be the highest at the midpoint, but the amount of sintering is rather suppressed at the center since the thermocapillary shear flow pulls Ag NP ink from the center to the both edges. As a result, the sintering of Ag NP happens at the sides that are marginally away from the midpoint as shown in the optical images, where the thermocapillary shear flow becomes sufficiently weak. The Ag NPs swept out from the trench, together with the effect of thermocapillary vortex flow [29,30], may also increase the concentration of Ag NP in the ink to create favorable condition for the sintering.

The laser scanning at higher laser power and lower scanning speed (corresponds to the bottom-left corner of Figure 3a) creates well-defined trenches in general that is necessary for the proposed patterning as confirmed from the high optical transmittance at the center of the scanning path. However, when the scanning speed is relatively low, (e.g., 0.1 mm/s, 0.2 W) it is observable that the Ag NPs are pushed away from the scanning path to create Ag NP depletion zone near the scanning path due to the longer exposure to the thermocapillary shear flow. This feature becomes more severe as the laser power increases further (0.1 mm/s, 0.3 W) as verifiable from the larger area subject to the higher optical transmittance. The laser parameters therefore have to be carefully controlled to exhibit high optical transmittance at the center with minimal effect to the surroundings (1 mm/s, 0.3 W). A closer look at the optical image of the optimum condition reveals that there exist fringe patterns near the laser scanned area, which are more clearly detectable in the reflection optical microscope image as shown in Figure 3b(i). Different reflective spectra presumably originate from the thin-film interference of Ag NP layer [31,32], and the fringe patterns substantiates that the thickness variation is introduced to the Ag NP layer due to the thermocapillary flow. Figure 3b(ii) is the optical image of the same sample under the identical illumination and magnification, but taken after being stored in the ambient condition for 10 min. It is noticeable that the spatial frequency of the fringe pattern is decreased, signifying that the Ag NP ink layer flattens over time while the Ag NP ink does not intrude into the trench during the flattening.

**Figure 4:**
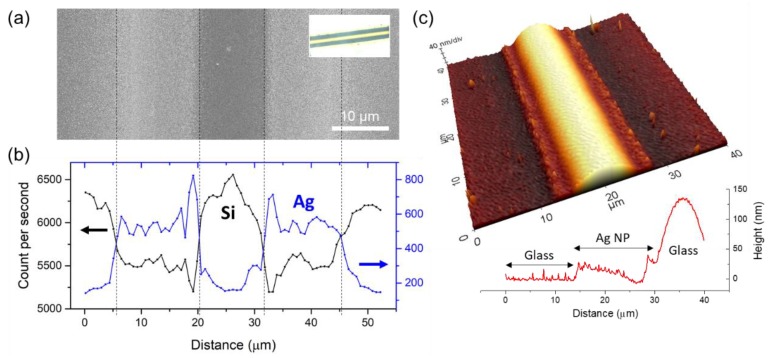
(**a**) Scanning Electron Microscope (SEM); (**b**) Energy Dispersive X-ray (EDX) and (**c**) Atomic Force Microscopy (AFM) measurement of the Ag NP layer which is cleaned with toluene immediately after the scanning process. The inset of (**a**) corresponds to its transmission optical microscope image. The 3D height profile in (**c**) is drawn at the scale of *X*:*Y*:*Z* = 1:1:30. The graph in (**c**) shows the height profile along the line perpendicular to the scanning path.

The sample after the scanning process is immediately cleaned with toluene and measured with SEM and AFM in order to investigate its microscopic properties further. The SEM image in Figure 4a together with its optical image (inset) approves that two distinct lines corresponding to the ridges are created symmetrically on both sides of the scanning path, and the EDX measurement along the line perpendicular to the scanning path (Figure 4b) confirms that these lines are indeed from the Ag NPs. While on the other hand, it is noticeable that the intensity of Ag element at the center is significantly lower and reaches similar level to the surroundings, indicating that the proposed method successfully pushes Ag NPs away from the scanning path. The intensity profile of the Si element is complementary to the Ag element in overall since the underlying glass layer, which is relevant to the Si intensity, is blocked by the presence of Ag layer above. Meanwhile, AFM measurement reveals rather interesting feature in its morphology that is not detectable in the SEM measurement. AFM measurement on 40 μm × 40 μm area in the vicinity of the scanning path shows that a consistent bump at 100 ~ 150 nm in height is created along the scanning path as shown in Figure 4c. Since this bump cannot be Ag as verified from the EDX measurement, we estimate that the feature is started from the underlying glass. Its smooth and spherical height profile (refers to the inset graph) also supports the theory that the bump is created by the laser-induced swelling of the glass [33,34,35]. The resultant morphology produces favorable condition for the proposed patterning since the bump may act as a barrier that prevents remerging of the Ag NP ink. The AFM profile of the typical void after the sintering through bulk heating of the entire sample can be found in Supporting Figure S1.

In the current experimental setting, the localized thermocapillary flow is generated by a focused laser spot the diameter of which is only at several micrometers. For the creation of voids with arbitrary geometrical features, the laser beam has to undergo a scanning procedure. The proposed technique can be expanded and applied to various types of geometrical features by incorporating more complex scanning. Supporting Figure S2 presents some of the results from our preliminary studies on the other types of voids with different geometrical features including holes, cylindrical pins and rectangular areas created through the hatching procedure. The voids at different sizes and shapes are generated by the proposed method, yet it is observable from the images that there exists remaining NPs in the voids to some extent. The scanning parameters—laser power, scanning speed, hatching distance, scanning path, etc.—for each case therefore have to be further optimized in order to achieve more refined results.

**Figure 5:**
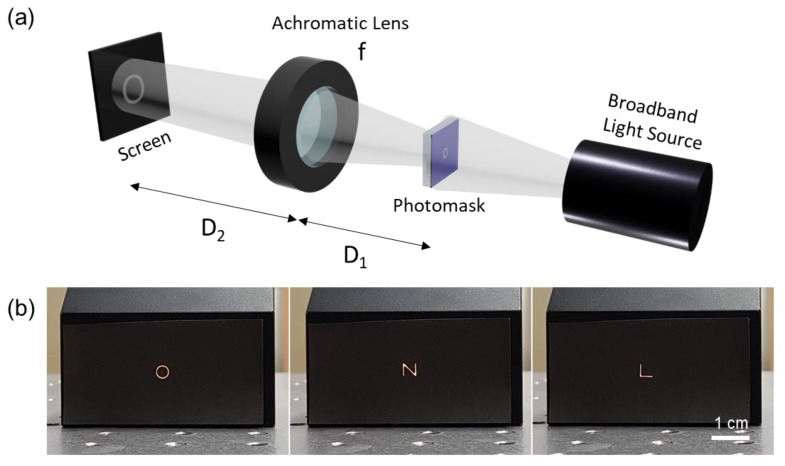
(**a**) Schematics for the optical setting for the reproduction of the void pattern created on the Ag NP layer by the proposed technique. (f = 100 mm, D_1_ = 150 mm, D_2_ = 300 mm); (**b**) Photographs of the Alphabetic letters (‘O’, ‘N’ and ‘L’) reproduced on the screen.

One of the applications that requires a metal layer with the voids at micrometer width is a dark-field photomask, which is manufactured by applying e-beam writer on a chrome mask blank in general. The proposed laser patterning enables fabrication of patterned metal layer that can play an analogous role as a dark-field photomask. Three different Alphabetic letters (‘O’, ‘N’ and ‘L’) are firstly patterned on Ag NP layer in 160 μm × 160 μm area on 26 mm × 26 mm sample size through the proposed method and bulk heated to transform the remaining Ag NPs into a continuous electrode layer. It is worth mentioning that the total time consumed for the scanning is less than a second, while the scanning time increases to ~10 min with the previous SLS process for the same photomask, assuming that the scanning speed is set to be 100 mm/s at 10 μm hatching distance. The total processing time however increases when the bulk heating time is considered in addition to the scanning time. To mimic the function of a photomask, the Alphabetic letters on the Ag NP layer are reproduced on a screen through a simple optical system which is schematically shown in Figure 5a. An achromatic lens with *f* = 100 mm is employed as a focusing lens to be compatible with the broadband light source, and the object distance (D_1_) and the image distance (D_2_) are set to be 150 mm and 300 mm respectively in order to reproduce the Alphabetic letters at 2× magnification according to the lens equation [31]. The resultant optical photographs are shown in Figure 5b, confirming that the Alphabetic letters are successfully reproduced on the screen.

## 4. Conclusions

The micropatterning of metal nanoparticle ink is reported in this study by harnessing the thermocapillary flow induced by the focused laser, which has been rather avoided in the relevant studies to date. The proposed technique can be an efficient complementary process for the previous SLS process for metal NP ink since these two processes can be conducted in the identical optical platform. The result is presented only with a specific Ag NP ink, however, we expect that the proposed method is compatible to wide range of metal NP inks as far as the temperature coefficient of surface tension is negative. In the same manner, the proposed method offers plenty of scope for expansion in terms of material selection, since the SLS process for metal NP ink has been successfully applied to the various types of materials including other metal nanoparticles [15,16,20] as well as metal-oxide nanoparticles [21,36,37] since it was first reported. We expect that the proposed technique can be particularly helpful for the manufacturing of NP-based metal layer composed of large metal area with sharp voids such as photomasks and current collectors for large-area energy devices. A current collector often does not require any patterning process, but specific current collectors, e.g., interdigitated electrodes [38], require specific voids which appears to be well compatible to the proposed technique.

Meanwhile, it is worth mentioning that the proposed technique has certain limitations, at least with the current optical setting. One of the limitations is the occurrence of depletion zone as shown in the Figure 3, although this can be minimized by careful control of laser scanning parameters. Such depletion zone has to be recovered before generating subsequent patterns, which increases the overall processing time. At the same time, the proposed technique incorporating scanning procedure is not efficient for overlapping patterns. An example can be found in Supporting Figure S3, where two scanning lines are intersecting each other in perpendicular directions. It is noticeable that the thermocapillary flow created by the second scanning results in the intrusion of Ag NP ink into the previously created trench. This result clearly shows the limitation of the proposed technique, but only under the current optical setting: it is expected that more advanced optical setting, which enables (spatial/temporal) dynamic beam shaping, such as digital micromirror device (DMD) and spatial light modulator (SLM) can expand available geometrical features to a great extent by eliminating the need for laser scanning procedure.

## Supplementary Materials

The following are available online at http://www.mdpi.com/2079-4991/8/9/645/s1, Figure S1: Transmission optical microscope image and AFM height profile of sintered Ag NP layer after applying the proposed method to generate a line pattern, Figure S2: Hatching schematics and the transmission optical microscope images of (**a**) holes at (i) *R*_max_ = 1.2 mm (ii) *R*_max_ = 0.8 mm (ii) *R*_max_ = 0.4 mm with *h* = 10 μm (**b**) cylindrical pins at (i) *L* = 0.5 mm (ii) *L* = 1.0 mm (iii) *L* = 1.5 mm with *D* = 10 μm and (**c**) rectangular area with *L* = 1.5 mm with *D* = 10 μm, Figure S3: Transmission optical microscope image of Ag NP layer after applying the proposed method overlapped in two perpendicular directions.
