# Supplementary material for: Micropatterning of Metal Nanoparticle Ink by Laser-Induced Thermocapillary Flow
Source: Nanomaterials (Basel). 2018 Aug 22;8(9):645. doi: 10.3390/nano8090645 (PMC6165030; doi:10.3390/nano8090645)
Supplement: Supplementary file 1 [file nanomaterials-08-00645-s001.zip › nanomaterials-336633-SI.pdf]

Micropatterning of Metal Nanoparticle Ink by Laser-Induced Thermocapillary Flow

Sewoong Park, Jinhyeong Kwon, Jaemook Lim, Wooseop Shin, Younggeun Lee, Habeom Lee, Hyun-Jong Kim, Seungyong Han, Junyeob Yeo, Seung Hwan Ko, and Sukjoon Hong

Supporting Information

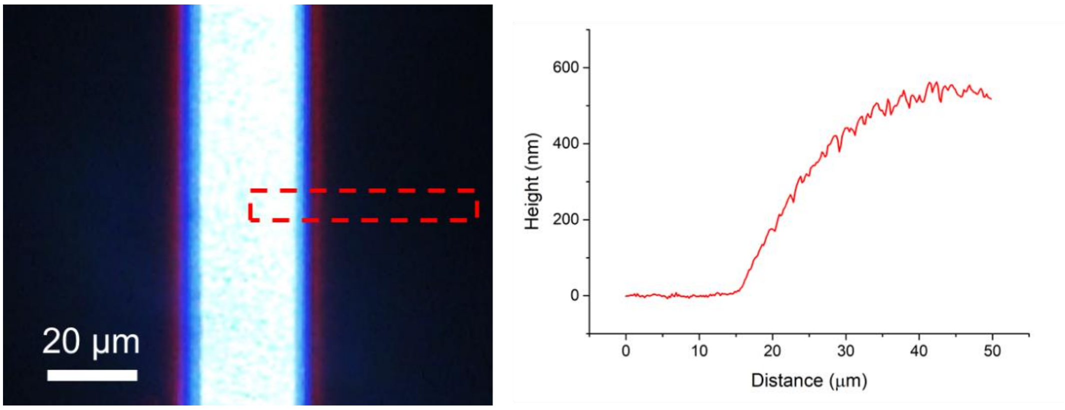

Figure S1. Transmission optical microscope image and AFM height profile of sintered Ag NP layer after applying the proposed method to generate a line pattern.

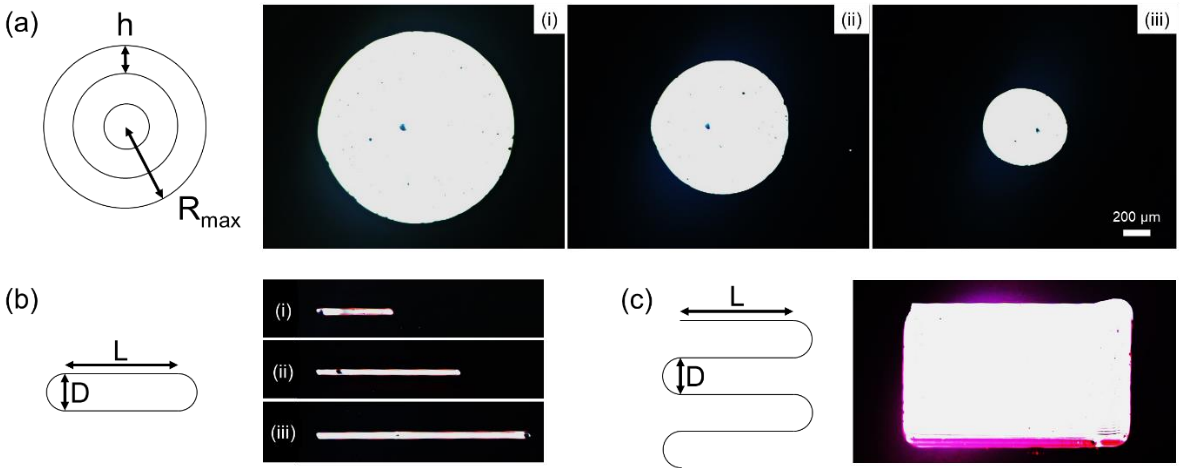

Figure S2. Hatching schematics and the transmission optical microscope images of (a) holes at (i) *R*max = 1.2 mm (ii) *R*max = 0.8 mm (ii) *R*max = 0.4 mm with *h* = 10 μm (b) cylindrical pins at (i) *L* = 0.5 mm (ii) *L* = 1.0 mm (iii) *L* = 1.5 mm with *D* = 10 μm and (c) rectangular area with *L* = 1.5 mm with *D* = 10 μm.

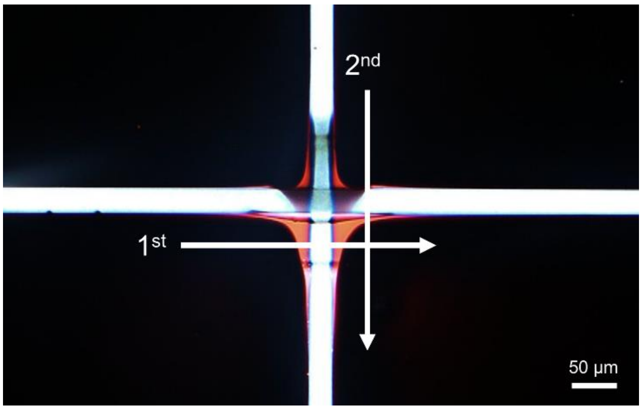

Figure S3. Transmission optical microscope image of Ag NP layer after applying the proposed method overlapped in two perpendicular directions.
